# Pre-Exposure to Moving Form Enhances Static Form Sensitivity

**DOI:** 10.1371/journal.pone.0008324

**Published:** 2009-12-17

**Authors:** Thomas S. A. Wallis, Mark A. Williams, Derek H. Arnold

**Affiliations:** 1 School of Psychology, The University of Queensland, St Lucia, Queensland, Australia; 2 Macquarie Centre for Cognitive Science, Macquarie University, Sydney, Australia; Rutgers University, United States of America

## Abstract

**Background:**

Motion-defined form can seem to persist briefly after motion ceases, before seeming to gradually disappear into the background. Here we investigate if this subjective persistence reflects a signal capable of improving objective measures of sensitivity to static form.

**Methodology/Principal Findings:**

We presented a sinusoidal modulation of luminance, masked by a background noise pattern. The sinusoidal luminance modulation was usually subjectively invisible when static, but visible when moving. We found that drifting then stopping the waveform resulted in a transient subjective persistence of the waveform in the static display. Observers' objective sensitivity to the position of the static waveform was also improved after viewing moving waveforms, compared to viewing static waveforms for a matched duration. This facilitation did not occur simply because movement provided more perspectives of the waveform, since performance following pre-exposure to scrambled animations did not match that following pre-exposure to smooth motion. Observers did not simply remember waveform positions at motion offset, since removing the waveform before testing reduced performance.

**Conclusions/Significance:**

Motion processing therefore interacts with subsequent static visual inputs in a way that can improve performance in objective sensitivity measures. We suggest that the brief subjective persistence of motion-defined forms that can occur after motion offsets is a consequence of the decay of a static form signal that has been transiently enhanced by motion processing.

## Introduction

A visual form that is camouflaged when stationary but revealed by motion can be said to be *motion-defined*. An interesting situation can ensue when a form is revealed by motion and then motion suddenly stops. Observers often experience a perceptual persistence, such that the motion-defined form remains subjectively visible for a brief interval in the absence of movement, before seeming to fade into the background and disappear from view [1], [2], [3], [4], [5], [6]. These transiently persisting forms do not subjectively appear to move, nor do they seem to persist if the entire display is removed [4].

Studies that have investigated motion-defined form persistence have typically used line drawings of animals and other objects masked by additional randomly positioned and oriented lines [2], [3], [4], [5], [6]. These studies have measured how the subjective persistence of motion-defined form is influenced by factors of interest. For example, the apparent duration of persistence is independent of attentional load and working memory constraints [3], but can be modulated by semantic information [6]. Implicit in these investigations is the assumption that the perceptual fading represents a gradual decay of a static visual form signal, rather than a bias to report forms seen previously.

If motion-defined form persistence reflects a transient motion-induced facilitation of static form perception that decays over time, one should be able to objectively measure the facilitation. Alternatively, if this behavior reflects a bias to report the presence of an object where it has recently been seen, no facilitation should be observed in objective measures of sensitivity. Our results demonstrate that pre-exposure to moving form can facilitate an objective measure of static form sensitivity.

## Results

**Figure 1:**
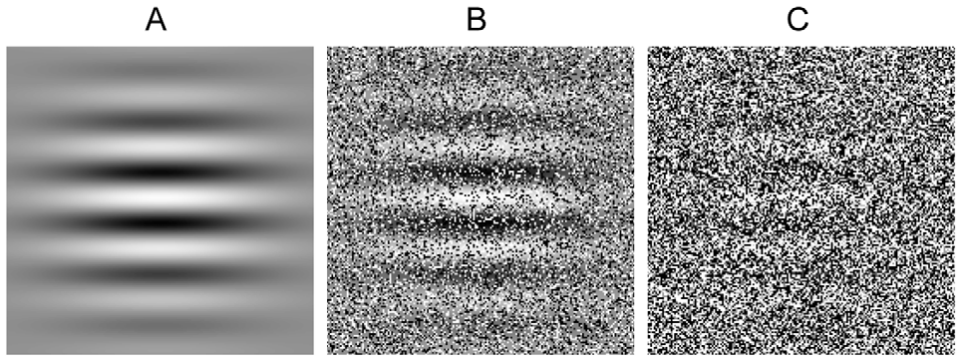
Illustration of “dot-view” stimulus generation. A) Target waveforms consisted of Gabor patterns or gratings. These were masked by replacing a proportion of the pixels depicting target waveforms (signal elements) with pixels depicting static white noise (noise elements). Signal elements can be thought of as windows, through which target waveforms can be seen. In this class of stimulus, the visibility of target waveforms can be adjusted by manipulating the proportion of signal elements to noise elements. B) Depiction of dot-view stimulus signal-to-noise ratio (SNR; proportion signal elements divided by proportion noise elements) 1.5. C) Depiction of dot-view stimulus SNR 0.33.

These experiments used a visual display that we refer to as a “dot-view” stimulus (see Figure 1, Methods, Movie S1 and Movie S2). Conceptually, this stimulus is very similar to multi-aperture displays [7] and slit-view displays, [8], [9], [10], [11]. The signal-to-noise ratio of the display was adjusted to make waveforms difficult to detect when stationary, but clearly visible when moving. The signal-to-noise ratios (0.33 in Experiment 1, 0.25 in Experiments 2 and 3) used in the reported Experiments were selected in order to avoid ceiling and floor effects for sensitivity judgments. Appropriate signal-to-noise ratios for this purpose were determined via a preliminary experiments.

### Experiment 1: Form Subjectively Persists After Movement

To confirm that our stimuli produced subjective form persistence, we used a procedure consistent with previous literature [3], [4], [5]. Target waveforms, visible through static windows interspersed amongst white noise (see Methods for further details), drifted up or down (determined at random on a trial-by-trial basis) for 1.5 seconds, then stopped. On half of the trials, the waveform remained (Form Stop condition), while on the other half the waveform pixels were spatially scrambled to produce a display with no coherent structure (Form Remove condition). Observers pressed a response button when “no coherent structure” remained in the static test patch (see Methods for further details). Thus, if waveforms seemed to fade instantly after motion offset, the response times in the two conditions should be equivalent. Alternatively, if static forms seem to persist before fading into the background, response times in the Form Stop condition will be longer.

**Figure 2:**
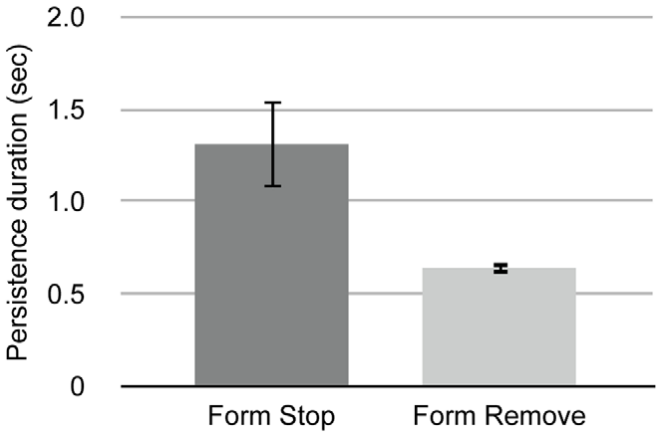
Subjective persistence of static form after motion offset. Times, post physical motion offset, at which observers reported that no coherent structure was visible in the display. When form was seen to move then stop, observers took longer to report that no coherent structure remained compared to when the coherent structure was physically removed at motion offset. Error bars depict +/− 1 s.e.m.

Observers signaled longer static form persistence in the Form Stop condition than in the Form Remove condition (Figure 2. [paired-samples t(4) = 2.95, p = 0.04]). Thus, our displays produce a subjective impression that motion-defined forms seem to persist briefly after motion offset before fading into the background of noise, consistent with previous literature [3], [4], [5]. This subjective impression was confirmed by all observers, on an informal basis, during this and subsequent experiments.

### Experiment 2: Motion Pre-Exposure Objectively Facilitates Subsequent Spatial Sensitivity

**Figure 3:**
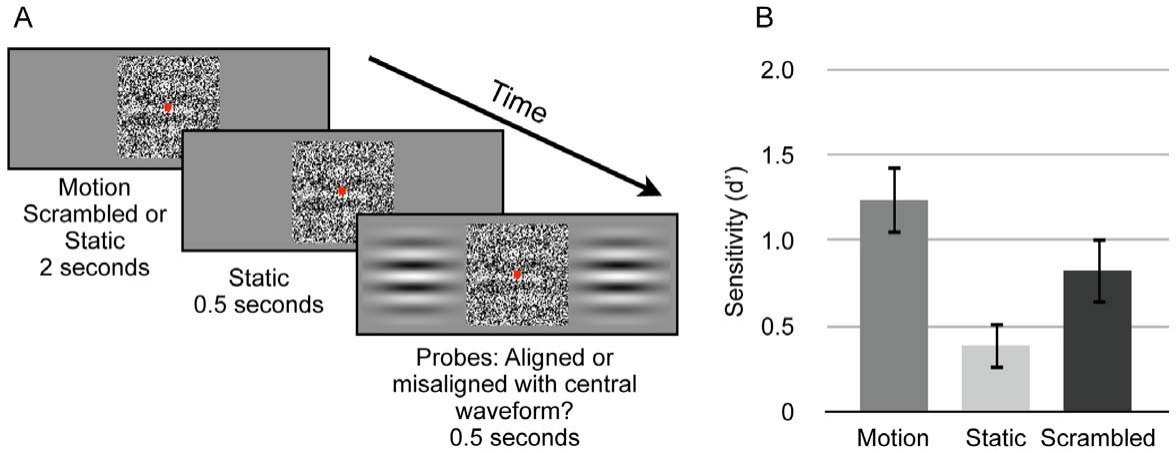
Experiment 2 procedure and alignment sensitivity for static forms. A) Depiction of procedure for Experiment 2. Observers viewed two second presentations of either smooth motion, scrambled motion, or of a static waveform. This was followed by a static inter-stimulus-interval (ISI), after which two adjacent probes were presented that contained waveforms either aligned or misaligned with the central test waveform. Observers were required to complete a forced choice task, indicating if the probe and central waveforms were aligned or misaligned. B) Alignment sensitivities (d′) for 11 observers. Error bars depict +/− 1 s.e.m.

We measured the effect of pre-exposure to a moving form on subsequent visual sensitivity for a static form using an alignment discrimination task (see Methods). A visual depiction of the experimental procedure is provided in Figure 3A.

A dot-view Gabor stimulus (see Methods) was presented centered on fixation (the test waveform), and either drifted coherently (Motion condition), remained stationary (Static condition), or movement frames were presented in a scrambled order (Scrambled condition) for two seconds. The test waveform then remained stationary for an additional 0.5 seconds. Respectively, these experimental conditions represent a situation identical to that producing motion-defined form persistence (in that a form is revealed by motion that then stops), a situation where the form is never revealed by motion, and a situation where the same number of perspectives of the form are presented in the absence of smooth movement.

Two unmasked probe waveforms were then presented to either side of the test waveform. On half the trials these had the same phase as the test waveform, such that the bars of the probes were aligned with the bars of the test waveform. On the other half of trials they were misaligned. On each trial observers made a forced-choice judgment, as to whether the test and probe waveforms were aligned or misaligned.

We analyzed responses using signal detection theory [12], [13] to yield estimates of objective sensitivity (*d′*) to alignments of the test and probe waveforms. As shown in Figure 3B, experimental conditions produced significantly different alignment sensitivities [within-subjects one-way ANOVA: F(2, 20) = 14.54, p<.0001]. There was a general bias (*c*) to report “aligned”, but this did not differ across experimental conditions [F(2, 20) = 2.96, p>.05].

We conducted follow-up comparisons for alignment sensitivities; all comparisons were two-tailed paired samples t-tests, and all significance values were compared to a Bonferroni-corrected alpha level for four comparisons (p<0.0125). Observers were more sensitive to the spatial position of the static test waveform after pre-exposure to coherent motion than after seeing the static waveform for a matched duration [t(10) = 5.24, p<0.0004]. Scrambled motion pre-exposure also facilitated sensitivity to spatial phase more than viewing the static display [t(10) = 4.7, p<0.001]. This indicates that seeing the form from a number of perspectives over time can improve performance relative to the static condition. Crucially, coherent motion pre-exposure facilitated sensitivity more than pre-exposure to scrambled motion [t(10) = 4.47, p<0.0012]. Thus, information derived from a coherent moving input can be used to facilitate subsequent visual judgments concerning static form. This result cannot simply be attributed to having view more perspectives of the forms, since scrambled motion did not facilitate spatial judgments equally.

### Experiment 3: Facilitation Is Not Based on Remembered Position at Motion Offset

**Figure 4:**
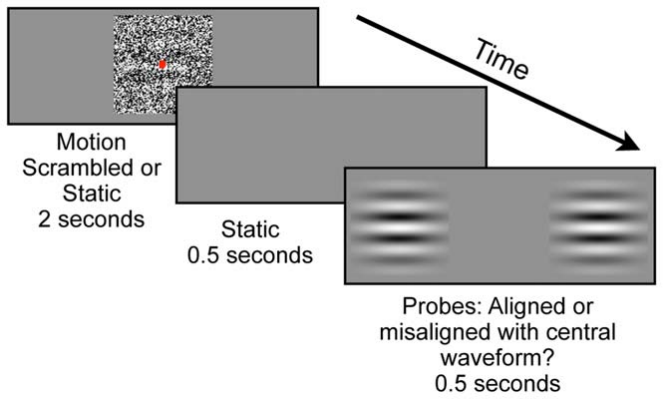
Experiment 3 procedure. A) Depiction of procedure for Experiment 3. Observers viewed two seconds of smooth motion, followed by a blank inter-stimulus-interval (ISI), after which two adjacent probes were presented that contained waveforms either aligned or misaligned with the previously visible central test waveform. Observers indicated if the probe waveforms were aligned or misaligned with the remembered test waveform.

It is plausible that observers in Experiment 2 responded by remembering the last perceived position of the moving waveform, and that the results of Experiment 2 had nothing to do with a facilitation of sensitivity to static input. To address this possibility, the same observers completed an additional condition, identical to the motion condition from Experiment 2 except that the test waveform was removed from the display during the inter-stimulus-interval (ISI) after motion offset (see Figure 4). Probes were presented together with the test waveform after the blank 0.5 second ISI. If the facilitation demonstrated in Experiment 2 was driven by the remembered position of the waveform at motion offset, rather than by an interaction involving subsequent static input, performance should be unaffected by the transient removal of the static test waveform.

This was not the case. The transient removal of the test waveform reduced performance [mean d′  = 0.54, s.e.m.  = 0.22] relative to the Motion condition from Experiment 2 [t(10) = 4.11, p<0.003]. Therefore, the motion-induced facilitation of spatial vision demonstrated in Experiment 2 is not due to observers remembering the position of the waveform at motion offset, but must be indicative of an interaction involving subsequent static visual input.

## Discussion

Previous studies have assumed that motion-defined form can briefly facilitate subsequent static form sensitivity, resulting in a transient perceptual persistence of the form after motion offset [2], [3], [4], [5], [6]. Our study suggests this assumption is sound. We have demonstrated that pre-exposure to a moving form can enhance performance in objective measures of sensitivity to alignments of static forms. This cannot simply be attributed to movement providing the observer with a greater number of perspectives of the form, as pre-exposure to scrambled animations did not result in an equivalent facilitation. Nor can the facilitation be attributed to the remembered position at motion offset, as the facilitation was eliminated by the removal of the test waveform at motion offset.

Our findings are compatible with previous literature investigating motion-defined forms. Pattern information can be resolved by interpolating spatial form information along the trajectory of motion [8], [14]. Motion-defined form and static luminance-defined form signals also have equivalent Vernier acuities [1]. In addition, motion-defined figural information seems to be more persistent than figural information defined by static luminance contrast [15]. In this last study, the authors showed that motion-defined figural information persisted across a static ISI, facilitating judgments involving motion-defined figural information in a subsequent display. Figural information defined by static luminance contrast also persisted across an interval in which the figural information was removed, facilitating judgments concerning subsequent static luminance-defined figures. However, the motion-defined information survived longer ISIs than did the luminance-defined information. Thus, previous literature suggests that motion-defined form is trajectory-dependent [8], [14], precise [1] and persistent [15], [16]. Our experiments suggest that this information can interact with analyses of subsequent static input, resulting in a transient facilitation of static form perception.

The suggestion motion-defined forms can interact with subsequent static input is consistent with broader literature on interactions between motion and form [17]. For instance, static form information can be used to improve motion perception [e.g. 18], [19], [20] and suitably-arranged motion vectors can imply 3D form where none exists [e.g. 21]. Human forms are also readily recognized from the coherent movement of points of light attached to the joints of human actors [22]. Of particular interest to the present research is the finding that biological form information, derived from point light motion, is integrated across intervals of several seconds [23]. This prolonged integration suggests that viewing biological motions should facilitate subsequent human static form detection in a similar manner to our results for simple waveforms.

Given that we have demonstrated an interaction wherein pre-exposure to moving forms can enhance the precision of spatial judgments concerning subsequent static inputs, it would be reasonable to ask whether pre-exposure to moving forms also facilitates subsequent static form detection. To assess this possibility with an objective measure (e.g. a 2 alternative forced-choice task), it would be necessary to contrast situations wherein static form signals are present and absent. This could be achieved by sometimes removing a static form signal after motion offset. However, there is a risk that observers could perform such a task on the basis of an offset transient magnitude, which would be greater in cases where a form signal is removed from the display relative to instances where it is not. Alternatively, stimuli could be completely removed and then, after variable ISIs, reintroduced at variable contrasts. However, this approach is also problematic as the facilitation of any moving-form mechanism might be disrupted by the offset of the form signal. Examining alignment sensitivity, as we have done, avoids these potential problems since motion offset transients are balanced across the critical aligned and misaligned conditions. Researchers interested in exploring any effect of motion pre-exposure on detection performance should be aware of these issues.

Our final experiment demonstrates that the removal of a static test stimulus can eliminate the motion pre-exposure facilitation of subsequent static form sensitivity. This is consistent with the absence of a fading form percept when the entire stimulus is removed [4]. These observations show that the sensory interactions underlying these effects will not elicit a perceptual experience of static forms in the absence of subsequent input. However, if the display is replaced by a locally dissimilar but globally complementary input at motion offset, a persistence illusion can still be experienced and enhanced BOLD activations in ventral brain regions can persist [5]. Thus, it would seem that the visual system can impose a global form signal onto structures that are discrepant at a fine spatial resolution, provided that the moving and subsequent static inputs are structurally similar at a coarse spatial resolution [5]. This suggests that the interactions underlying these perceptual effects operate at a coarse spatial resolution. The stimuli in the present study had a relatively low spatial frequency (see Methods). We speculate that the facilitation provided by motion pre-exposure would decline at finer spatial scales.

Several brain imaging (fMRI) studies [4], [5] suggest that perceptual persistence of motion-defined forms, post motion offset, are related to activations in brain regions along the ventral visual pathway, which are involved in object perception [e.g. 24,25,26]. Interestingly no correlated brain activity was found in the dorsal visual pathway (MT+) or in early visual areas (V1). A lack of correlation between V1 BOLD signals and the perceptual persistence of motion-defined forms is perhaps unsurprising. V1 responds to all visual input, so it would be expected to respond robustly to test stimuli regardless of whether the subject has been pre-exposed to moving form. Thus any differences in V1 BOLD signals might be subtle, particularly in comparison to brain regions selectively engaged for object recognition. We would suggest that while the perceptual persistence of motion-defined forms might be driven by ventral pathway activity [4], [5], this might still involve a modulation of V1 activity not readily apparent in BOLD measures. A role for early visual areas is certainly consistent with recent single cell recordings in macaque V2 concerning figure-ground segregation [27]. We plan on investigating this possibility using coherence measures of brain activity.

The present study shows that visual pattern information carried by motion-sensitive mechanisms can facilitate sensitivity to subsequent static input. We speculate that the perceptual fading of motion-defined form after motion offset reflects the decay of this transient facilitation over time.

## Materials and Methods

### General Methods

All experiments were approved by The University of Queensland School of Psychology ethics committee, and conducted according to the principles of the Declaration of Helsinki. Observers provided written informed consent prior to participating in the experiments. All observers had normal or corrected-to-normal vision.

Stimuli were generated using the CRS toolbox for Matlab, which controlled a ViSaGe (CRS) video card. Stimuli were displayed on a Sony Trinitron G420 monitor at a resolution of 1024 × 768 pixels and a refresh rate of 120 Hz. Observers viewed stimuli from a distance of 57 cm with their head placed in a chin rest.

### Dot-View Stimuli

To generate a dot-view stimulus, we start with a target stimulus (see Figure 1A). Pixels depicting the target stimulus are referred to as signal elements. A proportion of display pixels are then set randomly to black or white. These are referred to as noise elements. The attributes of noise elements are unchanging. However, the dot-view stimulus can be animated by modulating signal element attributes. Viewing this stimulus is akin to viewing the target stimulus through a sieve [7]. The visibility of targets in dot-view stimuli can be modulated by manipulating the ratio of signal to noise pixels (see Figure 1B, Figure 1C, Movie S1 and Movie S2). In the reported experiments, we chose a signal-to-noise ratio (SNR) at which target stimuli were subjectively at or near invisible when static, but easily seen when moving.

Target stimuli in all experiments consisted of sinusoidal modulations of luminance around a grey point (CIE 1931 *x* = 0.261 *y* = 0.264 *Y* = 60 cd/m^2^). The phase of the waveform was randomly determined at the start of a trial.

### Experiment 1

The target stimulus consisted of a sinusoidal luminance modulation subtending 6.5 by 6.5 degrees of visual angle (dva), presented to either the left or right of a fixation point (4.3 dva from fixation to the centre of the waveform). The waveform had a spatial frequency of 2 cycles per degree of visual angle (cpd), and was oriented horizontally. The Michelson contrast of the waveform was 0.3. Noise elements within the dot-view display (see Figure 1) had a Michelson contrast of 0.7 and subtended ∼0.04 dva. The SNR was 0.33.

The target stimulus waveform drifted upwards or downwards, determined at random on a trial-by-trial basis, at 8 Hz for 1.5 seconds. On half the trials the waveform then stopped moving (Form Stop condition). On the other half of the trials, after movement, the target stimulus was replaced by a scrambled version (Form Remove condition). Thus, the Form Remove test frame had the same average luminance and contrast as the Form Stop test frame, but contained no coherent waveform.

Observers indicated when no coherent structure was apparent in the display after motion offset by pressing a button. We recorded the time elapsed post motion offset until this response. Each condition was presented 30 times in a random order. Observers included two of the authors and three observers who were naïve as to the purpose of the experiment.

### Experiment 2

The target component of the dot-view stimulus was a horizontal sinusoidal luminance-modulated grating with a Michelson contrast of 0.25 and a spatial frequency of 0.75 cpd, windowed in a spatial Gaussian contrast envelope (sd of 1.6 dva). Noise elements within the dot-view stimulus subtended ∼0.07 by 0.07 dva and had a Michelson contrast of 1. The entire dot-view stimulus subtended 6.5 by 6.5 dva and was centered on a red fixation point. The SNR of the dot-view stimulus was 0.25.

At the start of each presentation, the target waveform was either drifted upwards or downwards (determined at random on a trial-by-trial basis) at 2 Hz (Motion condition), remained stationary (Static condition), or the frames of the motion condition were presented in a random order (Scrambled condition) for 2 seconds. This was followed by a 1 second presentation of a static target waveform. After 0.5 seconds, two probe waveforms were presented on either side of the central dot-view stimulus (centered 6.5 dva to the left and right of fixation). These remained on the screen, adjacent to the static dot-view stimulus, for 0.5 seconds, after which the entire display was blanked to the background grey.

Probe waveforms had the same spatial characteristics as the test waveform but were not presented in a dot-view display (see Figure 3A). In half the trials, probe waveforms had the same phase as the target waveform within the dot-view display (aligned). In other trials they were offset by 180 degrees of phase (misaligned). Observers were required to indicate whether probe and target waveforms were aligned or misaligned. Auditory feedback was provided.

Each run of trials consisted of 60 trials for each condition. Observers completed two runs-of-trials. Signal detection measures for each condition were thus calculated from 120 trials (60 aligned, 60 misaligned). Two of the authors and nine observers who were naive as to the experimental hypotheses participated in this experiment.

### Experiment 3

Details concerning Experiment 3 were the same as those for Experiment 2, with the following exceptions. The central dot-view stimulus was removed from the display 2 seconds into the trial. Thus observers experienced a coherently moving waveform for 2 seconds, followed by a 0.5 second blank ISI, followed by a 0.5 sec presentation of the two peripheral probe waveforms in the absence of the central dot-view stimulus. Observers were required to judge whether the probe waveforms were aligned with the last *remembered* position of the target waveform within the central dot-view stimulus. Each observer completed a single run of 120 trials. This experiment was completed after Experiment 2, so any improvement due to practice would enhance performance in this task relative to Experiment 2. However, the opposite result was obtained.

## Supporting Information

Movie S1 This movie depicts a “dot-view” waveform at a relatively high SNR.
(7.26 MB MOV)

Movie S2 This movie depicts a “dot-view” waveform at a lower SNR than the first demonstration. Depending on browser and screen performance, observers should be able to clearly make out the drifting waveform when animated. If the movie is manually stopped using the pause button, observers may notice that the form seems to persist for a short duration before fading into the background of noise.
(7.26 MB MOV)
